# The Effect of *SMN* Gene Dosage on ALS Risk and Disease Severity

**DOI:** 10.1002/ana.26009

**Published:** 2021-01-15

**Authors:** Matthieu Moisse, Ramona A. J. Zwamborn, Joke van Vugt, Rick van der Spek, Wouter van Rheenen, Brendan Kenna, Kristel Van Eijk, Kevin Kenna, Philippe Corcia, Philippe Couratier, Patrick Vourc'h, Orla Hardiman, Russell McLaughin, Marc Gotkine, Vivian Drory, Nicola Ticozzi, Vincenzo Silani, Mamede de Carvalho, Jesús S. Mora Pardina, Monica Povedano, Peter M. Andersen, Markus Weber, Nazli A. Başak, Xiao Chen, Michael A. Eberle, Ammar Al‐Chalabi, Chris Shaw, Pamela J. Shaw, Karen E. Morrison, John E. Landers, Jonathan D. Glass, Wim Robberecht, Michael van Es, Leonard van den Berg, Jan Veldink, Philip Van Damme, Project MinE Sequencing Consortium

**Affiliations:** ^1^ Departments of Neurosciences, Experimental Neurology, and Leuven Brain Institute (LBI) KU Leuven – University of Leuven Leuven Belgium; ^2^ Laboratory of Neurobiology VIB, Center for Brain and Disease Research Leuven Belgium; ^3^ Department of Neurology UMC Utrecht Brain Center, Utrecht University Utrecht The Netherlands; ^4^ Centre SLA, CHRU de Tours Tours France; ^5^ UMR 1253, iBrain, Université de Tours, Inserm Tours France; ^6^ Academic Unit of Neurology Trinity College Dublin, Trinity Biomedical Sciences Institute Dublin Republic of Ireland; ^7^ Complex Trait Genomics Laboratory Smurfit Institute of Genetics, Trinity College Dublin Dublin Republic of Ireland; ^8^ The Agnes Ginges Center for Human Neurogenetics Hadassah‐Hebrew University Medical Center Jerusalem Israel; ^9^ Department of Neurology Tel‐Aviv Sourasky Medical Centre Tel Aviv Israel; ^10^ Department of Neurology, Stroke Unit and Laboratory of Neuroscience IRCCS Istituto Auxologico Italiano Milano Italy; ^11^ Department of Pathophysiology and Transplantation “Dino Ferrari” Center, Università degli Studi di Milano Milan Italy; ^12^ Instituto de Fisiologia, Instituto de Medicina Molecular, Faculdade de Medicina Universidade de Lisboa Lisbon Portugal; ^13^ ALS Unit Hospital San Rafael Madrid Spain; ^14^ Servei de Neurologia, HUB‐IDIBELL Barcelona Spain; ^15^ Department of Clinical Science, Neurosciences Umeå University Umeå Sweden; ^16^ Neuromuscular Diseases Unit/ALS Clinic St. Gallen Switzerland; ^17^ Koç University, School of Medicine, KUTTAM‐NDAL Istanbul Turkey; ^18^ Illumina Inc. San Diego CA; ^19^ Department of Basic and Clinical Neuroscience Maurice Wohl Clinical Neuroscience Institute, King's College London London UK; ^20^ Sheffield Institute for Translational Neuroscience (SITraN) University of Sheffield Sheffield UK; ^21^ School of Medicine, Dentistry, and Biomedical Sciences Queen's University Belfast Belfast UK; ^22^ Department of Neurology University of Massachusetts Medical School Worcester MA; ^23^ Department Neurology Emory University School of Medicine Atlanta GA; ^24^ Department of Neurology University Hospitals Leuven Leuven Belgium; ^25^ Project MinE Sequencing Consortium Utrecht The Netherlands

## Abstract

**Objective:**

The role of the survival of motor neuron (*SMN*) gene in amyotrophic lateral sclerosis (ALS) is unclear, with several conflicting reports. A decisive result on this topic is needed, given that treatment options are available now for *SMN* deficiency.

**Methods:**

In this largest multicenter case control study to evaluate the effect of *SMN1* and *SMN2* copy numbers in ALS, we used whole genome sequencing data from Project MinE data freeze 2. *SMN* copy numbers of 6,375 patients with ALS and 2,412 controls were called from whole genome sequencing data, and the reliability of the calls was tested with multiplex ligation‐dependent probe amplification data.

**Results:**

The copy number distribution of *SMN1* and *SMN2* between cases and controls did not show any statistical differences (binomial multivariate logistic regression *SMN1 p* = 0.54 and *SMN2 p* = 0.49). In addition, the copy number of *SMN* did not associate with patient survival (Royston‐Parmar; *SMN1 p* = 0.78 and *SMN2 p* = 0.23) or age at onset (Royston‐Parmar; *SMN1 p* = 0.75 and *SMN2 p* = 0.63).

**Interpretation:**

In our well‐powered study, there was no association of SMN1 or SMN2 copy numbers with the risk of ALS or ALS disease severity. This suggests that changing SMN protein levels in the physiological range may not modify ALS disease course. This is an important finding in the light of emerging therapies targeted at SMN deficiencies. ANN NEUROL 2021;89:686–697

Amyotrophic lateral sclerosis (ALS) and spinal muscular atrophy (SMA) are both motor neuron disorders leading to progressive muscle weakness and death of patients, mostly due to respiratory failure.^1^, ^2^

ALS is an adult‐onset disease with an estimated lifetime risk of 1 in 400. Approximately 50% of patients die within 3 to 5 years after onset, but there is a high level of variability between patients in age at onset and progression rate, with 5% of the patients surviving more than 10 years.^3^, ^4^ Patients are typically categorized into familial ALS representing 10 to 20% of patients and sporadic ALS. Genetically, ~ 66% of familial and ~ 10% sporadic patients are explained by a mutation in one of the ~ 25 genes that have been associated with ALS, with mutations in *SOD1*, *TARDBP*, *FUS*, and *C9orf72* being the most common causes.^1^ To date, several clinical and genetic factors have been put forward as disease modifiers, for example, sex, age at onset, site of onset, diagnostic delay, presence of frontotemporal dementia, *C9orf72* repeat expansions and SNPs in or near *UNC13A*, and CAMTA1.^5^ In addition, copy number (CN) variation in *SMN*, the gene that is deleted in childhood onset SMA, has been extensively studied as disease modifier with many conflicting reports.^6^, ^7^, ^8^, ^9^, ^10^, ^11^, ^12^, ^13^, ^14^, ^15^

SMA is a monogenic disease usually caused by a homozygous loss of *SMN1* and modulated by the number of *SMN2* copies present.^16^ In ~ 95% of the cases, both copies of *SMN1* are missing or affected by a gene conversion, the remaining ~ 5% of the patients have a mutation on their remaining copy of *SMN1*.^17^ The *SMN* genes are located on q13.2 of chromosome 5, a locus that underwent an inverted duplication of about 500 kb.^18^ *SMN2* is almost identical to *SMN1*, except for a few point mutations and small insertions and deletions, one of which is located at the splice junction of exon 7 and causes exon 7 skipping.^18^ As a consequence, *SMN2* mainly codes for a non‐fully functional SMN protein.^16^ Recently, 3 treatment options became available that aim to increase full length functional SMN protein levels. The first one is a multi‐dose antisense oligonucleotide therapy named nusinersen, the second is the single dose gene replacement therapy onasemnogene abeparvovec, and the third option is experimental compound therapies, aiming to increase the expression of the SMN locus.^19^, ^20^ Nusinersen works through blocking the binding of heterogeneous nuclear ribonucleoproteins (hnRNPs) at a splicing silencer element, named ISS‐N1, leading to exon 7 inclusion and increases full length SMN protein levels.^2^, ^19^ Onasemnogene abeparvovec adds a functional copy of *SMN1* to the genome using a self‐complementary adeno‐associated viral serotype 9, increasing the SMN protein levels.^20^

These recent therapeutic breakthroughs in SMA could have large consequences for ALS as well, if a convincing role for *SMN* in ALS could be established.

Previous studies investigating the effect of the CNs of *SMN* on disease risk and progression of ALS have reported conflicting results.^6^, ^7^, ^8^, ^9^, ^10^, ^11^, ^12^, ^13^, ^14^, ^15^ According to some studies, loss of *SMN1* is associated with ALS risk,^8^, ^9^, ^12^ whereas others found that duplication of *SMN1* associated with ALS risks.^6^, ^7^, ^11^, ^12^, ^13^ Others did not find any association between ALS and the number of *SMN1* copies.^10^, ^14^, ^15^ Likewise for *SMN2*, some found a homozygous deletion to be protective,^8^ whereas others found the deletion to be more frequent in ALS and reducing the survival time of these patients.^9^, ^10^, ^15^ In addition, here, several other publications failed to find any association.^6^, ^7^, ^11^, ^12^, ^13^, ^14^

In this large study, we aimed to use the whole genome sequencing (WGS) data from Project MinE, which contains 6,375 patients with ALS and 2,412 controls to evaluate the effect of *SMN* CNs in the context of ALS risk and clinical phenotype.^21^ This is the largest ALS cohort in which *SMN* genes have been analyzed in the hope to find a definitive answer.

## Methods

### *Experimental Design*

This WGS case–control study uses data freeze 2 from Project MinE, which contains a total of 9,600 whole genomes sequencing data from ALS cases and age‐matched and sex‐matched control samples.^21^ Samples were mostly of people of European descent and collected from 17 centers across 13 countries: Belgium, Switzerland, Spain, France, United Kingdom, Ireland, Israel, Italy, The Netherlands, Portugal, Sweden, Turkey, and the United States.

Patients were diagnosed with ALS in their respective centers, mainly using the El‐Escorial criteria. Clinical information was collected from each center and centrally harmonized and passed though quality control. For patients who are still alive, a survival update was requested on a yearly basis. All participants signed an informed consent at their respective centers. This study was approved by the respective ethics committees of the participating centers.

### *Sequencing, Variant Calling, and Quality Control*

The first batch of 2,250 cases and control samples were sequenced on the Illumina HiSeq 2000 platform. All remaining 7,350 cases and controls were sequenced on the Illumina HiSeq X platform. All samples were sequenced to ~ 35X coverage with 100 bp reads and ~ 25X coverage with 150 bp reads for the HiSeq 2000 and HiSeq X, respectively. Both sequencing sets used polymerase chain reaction (PCR)‐free library preparation. Samples were also genotyped on the Illumina 2.5 M array. Sequencing data were then aligned to GRCh37 using the iSAAC Aligner, and variants called using the iSAAC variant caller; both the aligner and caller are standard to Illumina's aligning and calling pipeline.

Per individual, gVCFs were merged using the Illumina gvcfgenotyper tool version 2018.10.15 (https://github.com/Illumina/gvcfgenotyper). Sites with a genotype quality (GQ) < 10 were set to missing and single nucleotide variations (SNVs) and indels with quality (QUAL) scores < 20 and < 30, respectively, were removed. Biological sex was inferred from the average coverage of chrY and chrX compared with the average coverage of the autosomal chromosomes.

Next sample‐level quality control was performed using the following filters at sample level: (1) more than 2% of the variant missing, (2) abnormal value of the autosomal homozygous genotype score calculated by Plink 1.9 |F| > 0.10, (3) aberrant transition/transversion ratio, defined as ±6 standard deviations (SDs), (4) aberrant number of variants, defined as ±6 SDs, (5) aberrant number of singletons, defined as ±6 SDs, (6) aberrant number of indels, defined as ±6 SDs, and (7) an inconsistency between the inferred and reported sex.

Variants in the merged vcf file were first decomposed and normalized using the corresponding commands in vt (version 2015.11.10) and then annotated by VEP (version 96), information from public databases dbNSFP (version 3.5a), dbscSNV (version 1.1), ExAC (version 0.3), gnomAD exome (version 2.1), ESP (ESP6500SI‐V2), 1,000 genomes (phase 3), dbsnp (version 151), and clinvar (version 20190513) were added using vcfanno (version 0.3.1). Annotated vcf files were loaded into gemini (version 0.30.1) and filtered for exonic variants, excluding synonymous variants not near splice sites, with an allele frequency of maximum 2%. Resulting variants in *SOD1*, *FUS*, and *TARDBP* were further inspected using the ACMG guidelines on the varsome platform.^22^ Pathogenic, likely pathogenic, and variants of unknown significance with some evidence toward pathogenicity were retained for further analysis. *C9ORF72* expansions were detected using Expansion Hunter (version 3.1.2) from Illumina.^23^

### *SMN Calling*

To call the CNs of *SMN* from WGS data we used the recently published SMNCopyNumberCaller (SMNCNC) tool from Illumina.^24^ In brief, this method uses read depth information at the 8 loci in the *SMN1* and *SMN2* genes to determine the consensus CN of both genes (coordinates against hg19): site number 7 (intron 6, SMN1 chr5‐70246320‐G, SMN2 chr5‐69370895‐A), site number 8 (intron 6, SMN1 chr5‐70246793‐G, SMN2 chr5‐69371368‐A), site number 10 (intron 6, SMN1 chr5‐70247219‐G, SMN2 chr5‐69371799‐A), site number 11 (intron 6, SMN1 chr5‐70247290‐T, SMN2 chr5‐69371870‐C), site number 12 (intron 6, SMN1 chr5‐70247724‐G, SMN2 chr5‐69372304‐A), site number 13 (Splice site variant in exon 7, SMN1 chr5‐70247773‐C, SMN2 chr5‐69372353‐T), site number 14 (intron7, SMN1 chr5‐70247921‐A, SMN2 chr5‐69372501‐G), and site number 15 (intron7, SMN1 chr5‐70248036‐A, SMN2 chr5‐69372616‐G). Wet lab validation was performed using multiplexed ligation‐dependent probe amplification (MLPA) using standard protocols as described by Blauw et al.^7^

### *Power Analysis*

Power calculations were performed for the survival and onset analysis using the powerCT function of the R package powerSurvEpi (version 0.1.0), with a *p* value threshold of 0.05.

### *Statistical Analysis*

To test the independence of the *SMN* CN distributions between cohorts we used a 2‐sided asymptotic generalized Pearson Chi‐Squared test (gχ^2^) provided by the chisq_test method of the R package coin (version 1.3–1), with *SMN* CN as ordinal and cohort as nominal. When comparing the *SMN* CN distribution of only two cohorts a 2‐sided asymptotic linear‐by‐linear (lbl) association test was used, implemented by the same chisq_test method.

For the corrected risk analysis, a 2‐sided binomial multivariate logistic regression was performed using the glm function from the R stats package. *SMN* CN was added to the model as a categorical variable with baseline a CN of 2, while correcting for the following terms: sex, sequencing technology, cohort, the first 20 HapMap projected principal components (PCs), and the mutation status of *C9orf72*, *SOD1*, *FUS*, and *TARDBP*.

Cox survival analysis was performed using the coxph function of the R package survival (version 3.1–8). Flexible survival regression using the Royston‐Parmar (RP) spline model was performed using the flexsurvspline function from the R package flexsurv (version 1.1.1). When assessing the effect of *SMN* CN on survival *SMN* CN was treated as a categorical variable with baseline a CN of 2. Survival analyses were corrected for age at onset, sex, site of onset, *C9orf72* expansion status, sequencing technology, cohort, and the first 20 HapMap projected PCs. Onset analyses were corrected for sex, sequencing technology, cohort, and the first 20 HapMap projected PCs.

Meta‐analysis of the individual cohort was performed using the meta (version 4.13–0) and metafor (version 2.4–0) R packages. The same covariates were used as in the risk, survival, and onset analysis, but only the first 5 HapMap projected PCs were used.

## Results

### *Validating SMN Calls from WGS Data*

**FIGURE 1:**
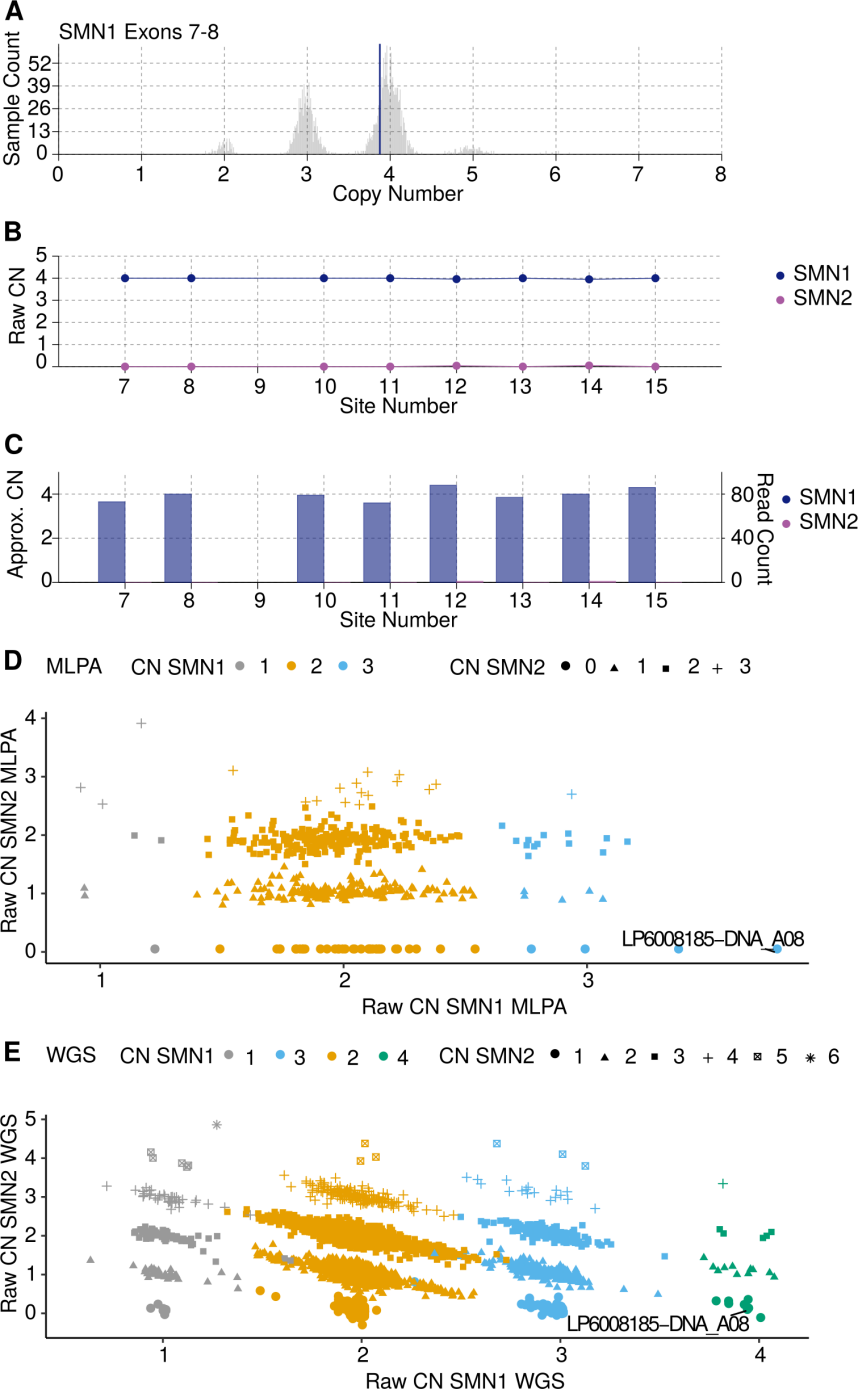
Raw data plots for discordant MLPA call. MPLA called 3 CNs for *SMN1* in sample LP6008185‐DNA_A08, whereas SMNCNC called 4 CNs for *SMN1*. Closer inspection revealed SMNCNC to be correct. (A) Raw CN of full length *SMN1* estimated by exon 7 and 8, histogram indicating the estimated CN of a large control cohort, vertical blue line indicates the discordant sample. (B) *SMN1* and *SMN2* raw CN values at the 8 loci used to determine the consensus CN. (C) *SMN1* and *SMN2* CNs normalized against coverage depth at 8 the loci. (D) Scatterplot of the raw CN calls by MLPA, on the x‐axis raw CN for *SMN1*, on the y‐axis raw CN for *SMN2*, discordant sample indicated with a line. (E) Scatter plot of the raw CN calls by SMNCNC, on the x‐axis raw CN for SMN1, and on the y‐axis raw CN for SMN2, discordant sample indicated with a line. CN = copy number; MLPA = multiplexed ligation‐dependent probe amplification; SMN = survival of motor neuron; SMNCNC = SMNCopyNumberCaller; WGS = whole genome sequencing.

**FIGURE 2:**
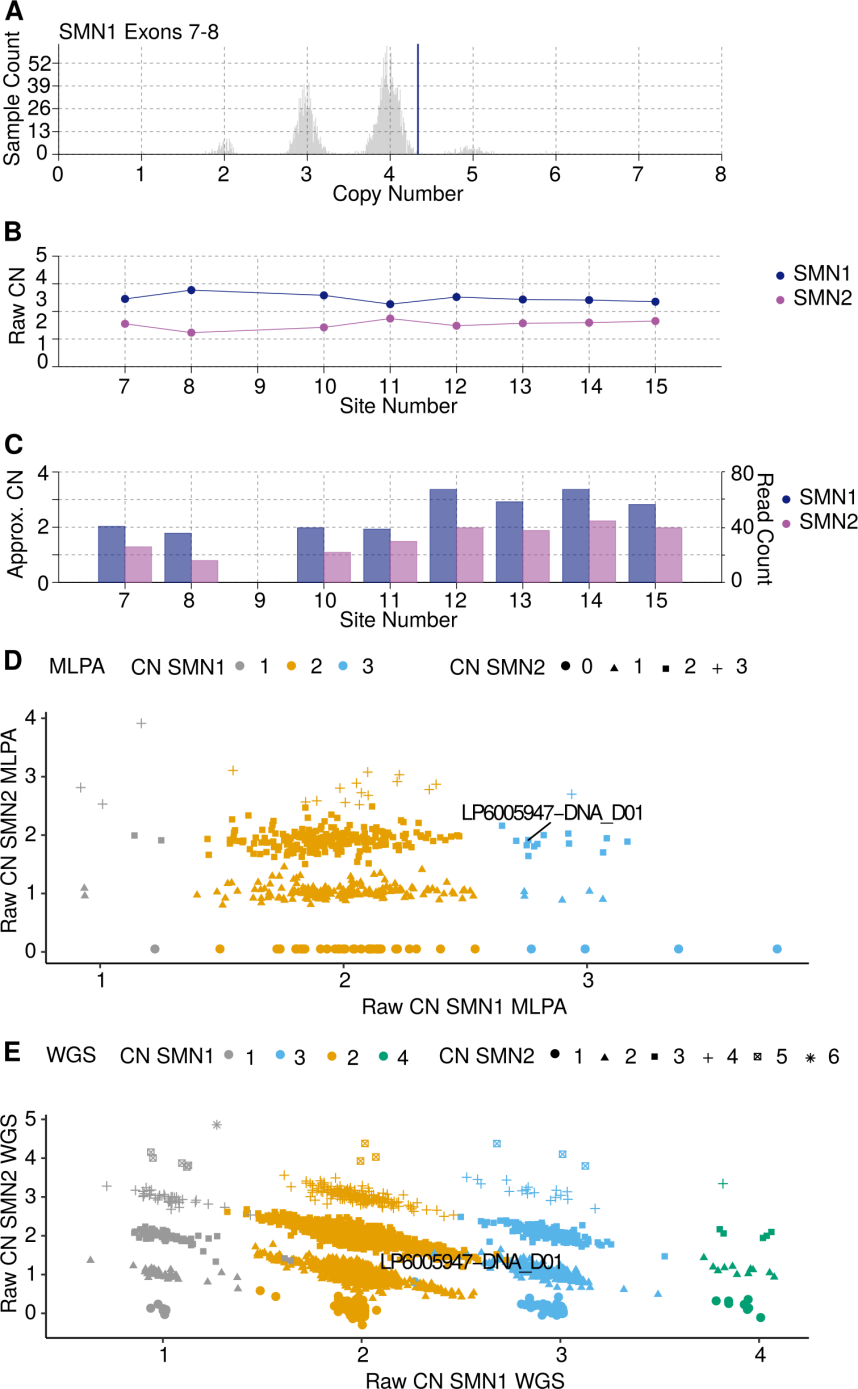
Raw data plots for discordant SMNCNC call. MPLA called 3 CNs for *SMN1* in sample LP6005947‐DNA_D01, whereas SMNCNC called 2 CNs for *SMN1*. Closer inspection revealed MLPA to be correct. (A) Raw CN of full length SMN1 estimated by exon 7 and 8, histogram indicating the estimated CN of a large control cohort, vertical blue line indicates the discordant sample. (B) *SMN1* and *SMN2* raw CN values at the 8 loci used to determine the consensus CN. (C) *SMN1* and *SMN2* CN normalized against coverage depth at the 8 loci. (D) Scatterplot of the raw CN calls by MLPA, on the x‐axis raw CN for *SMN1*, and on the y‐axis raw CN for *SMN2*, discordant sample indicated with a line. (E) Scatter plot of the raw CN calls by SMNCNC, on the x‐axis raw CN for SMN1, and on the y‐axis raw CN for SMN2, discordant sample indicated with a line. CN = copy number; MLPA = multiplexed ligation‐dependent probe amplification; SMN = survival of motor neuron; SMNCNC = SMNCopyNumberCaller; WGS = whole genome sequencing.

We used 2,412 controls and 6,375 cases that passed the QC metric and had sufficient clinical information (Table 1 and Table S1). For these samples, we ran SMNCNC to estimate the CN of *SMN1* and *SMN2*. For 475 of our samples, we also had *SMN* CNs available from MLPA. We excluded samples where the MLPA results for exon 7 and exon 8 disagreed with each other. The concordance between the MLPA results of exon 7 and exon 8 was 89.3% for SMN1 and 97.9% for SMN2. Excluding discordant samples resulted in a set of 413 samples. This set was then further compared with the results of SMNCNC, with a concordance of 99.3% and 99.5% for *SMN1* and *SMN2*, respectively, which is similar to previous findings (Figs 1 and 2).^24^

**TABLE 1:** Phenotype Information of the MinE Participants

Project MinE	ALS (%)		Control (%)	
	6375	(72.55)	2412	(27.45)
**Sex**				
Male	3,815	(59.84)	1,272	(52.74)
**C9orf72 status**				
Expanded	377	(5.91)	7	(0.29)
**Onset**				
Spinal	4,226	(66.29)		
Bulbar	1,734	(27.2)		
Generalized	221	(3.47)		
Thoracic/respiratory	112	(1.76)		
FTD	5	(0.08)		

ALS = amyotrophic lateral sclerosis; FTD = frontotemporal dementia.

### ****SMN**** ***CNs as a Risk Factor for ALS***

**FIGURE 3:**
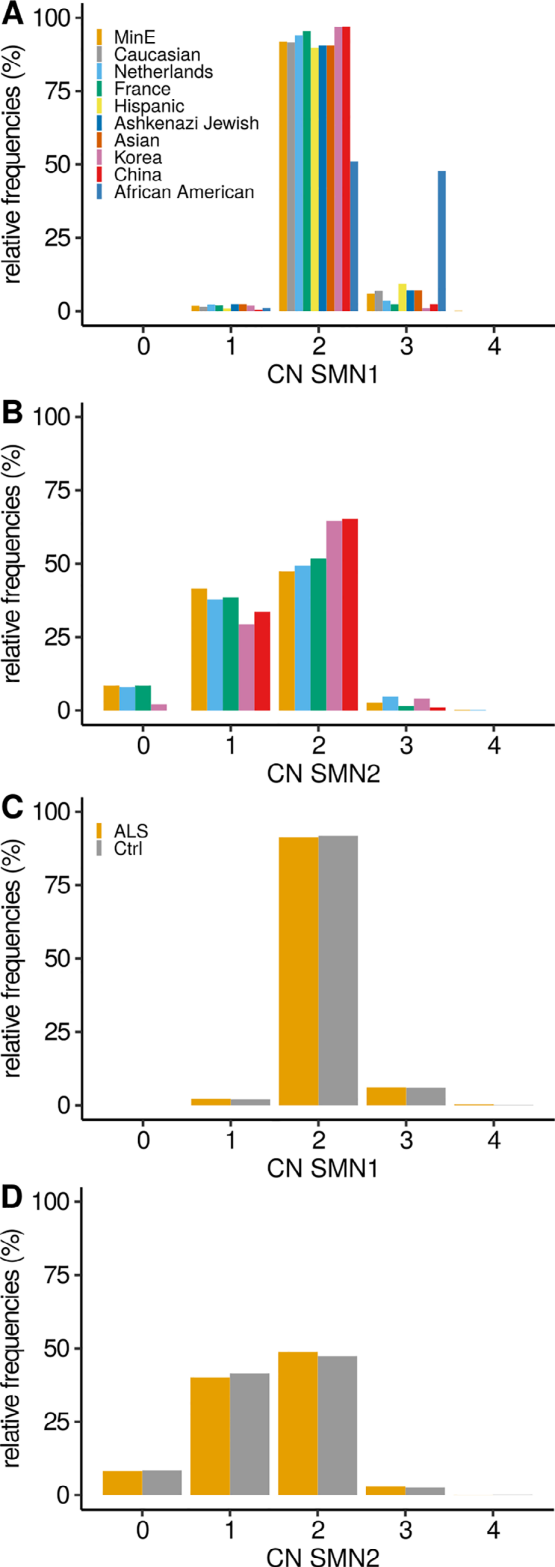
Frequency of the CNs of the *SMN1* and 2 genes. Frequency of (A) *SMN1* and (B) SMN2 copy number in control individuals of this study (MinE), Feng Y. et al. (White, Hispanic, Ashkenazi Jewish, Asian, and African American), Blauw HM. et al. (The Netherlands), Corcia P. et al. (France), Yoon S. et al. (Korea), and Fang P. et al. (China). Comparisons of the different cohort toward this study for *SMN1* (lbl *p* value): White (0.16), Netherlands (3.2 × 10^‐3^), France (1.3 × 10^‐3^), Hispanic (3.4 × 10^‐5^), Ashkenazi Jewish (0.20), Asian (0.83), Korea (0.07), China (0.25), and African American (<1 × 10^‐16^); and *SMN2*: Netherlands (1.1 × 10^‐2^), France (0.63), Korea (2.3 × 10^‐4^), and China (7.6 × 10^‐6^). Frequency of (C) *SMN1* and (D) *SMN2* copy numbers in patients with ALS and control individuals in this study. ALS = amyotrophic lateral sclerosis; CN = copy number; lbl = linear‐by‐linear.

**FIGURE 4:**
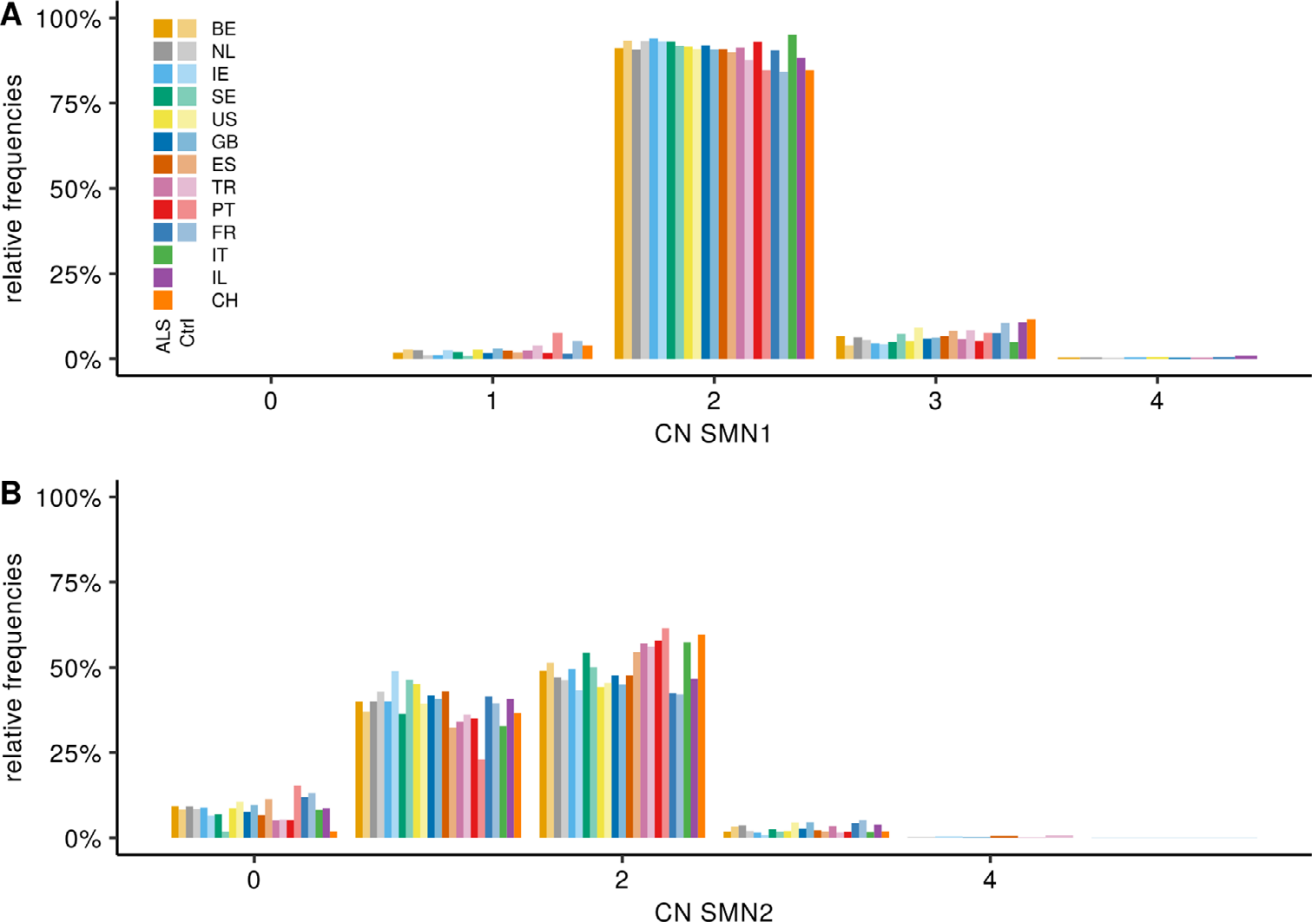
SMN frequencies by country. Frequency of *SMN1* (A) and *SMN2* (B) CN in ALS patients and control individuals in this study by cohort. Lighter colors indicate controls. Comparisons of the different ALS cohort toward the Turkish cohort for *SMN1* (lbl *p* value): BE (0.41), NL (0.75), IE (0.86), SE (0.67), US (0.79), GB (0.55), ES (0.96), PT (0.90), FR (0.21), IT (0.83), IL (1.1 × 10^‐2^) and CH (0.42); and SMN2: BE (7.1 × 10^‐5^), NL (2.5e‐5), IE (1.6 × 10^‐4^), SE (0.19), US (0.4.0 × 10^‐6^), GB (9.9 × 10^‐5^), ES (5.9 × 10^‐3^), PT (0.73), FR (2.3 × 10^‐4^), IT (0.44), IL (0.05), and CH (0.81). ALS = amyotrophic lateral sclerosis; BE = Belgium; CH = Switzerland; CN = copy number; GB = United Kingdom; ES = Spain; FR = France; IE = Ireland; IL = Israel; IT = Italy; lbl = linear‐by‐linear; NL = The Netherlands; PT = Portugal; SE = Sweden; SMN = survival of motor neuron; TR = Turkey; US = United States.

To investigate if our *SMN* CN estimates can be used in a risk factor analysis, we first compared the frequency of *SMN* copies in our controls to published literature (Fig 3A, B, and Table S2).^7^, ^12^, ^25^, ^26^, ^27^ To assess the CN distributions across multiple groups we used a gχ^2^ test, whereas we used the lbl test when comparing the distribution between the 2 groups. Assessing the *SMN1* and *SMN2* CN distribution across the different control populations revealed geographic differences, as previously reported (*SMN1*; gχ^2^ *p* < 2.2 × 10^‐16^; *SMN2* gχ^2^ *p* = 5.1 × 10^‐7^).^27^ When comparing our *SMN1* CN distribution to a Caucasian population of 2,175 individuals we did not find any difference (lbl *p* = 0.16; see Fig 3A), confirming that our control population is representative.^27^ Within the control samples of Project MinE no difference in *SMN1* or *SMN2* CN frequency was observed between the cohorts (*SMN1*; gχ^2^ *p* = 0.45; *SMN2*; gχ^2^ *p* = 0.53; Fig 4 and Tables S3 and S4).

When looking at *SMN* CN frequency in patients with ALS of published cohorts and our own we do observe a significant difference for *SMN1* CN (gχ^2^ *p* = 7.3 × 10^‐4^) but not for *SMN2* CN (gχ^2^ *p* = 0.10). Within Project MinE no differences between cohorts for *SMN1* CN (gχ^2^ *p* = 0.52) were observed, whereas for *SMN2* differences were observed (gχ^2^ *p* = 4.9 × 10^‐4^), in the Turkish cohort (see Fig 4B and Tables S3 and S4).

Comparing *SMN1* frequency between cases and controls within Project MinE revealed no significant differences (lbl *p* = 0.54), similarly *SMN2* also did not show any differences (lbl *p* = 0.23; see Fig 3C, D). Using a binomial logistic regression, we performed a corrected case control risk analysis for the *SMN* CN. Similarly, we did not observe any significant differences for the *SMN* CN status between cases and controls (binomial multivariate logistic regression; *SMN1 p* = 0.54 and *SMN2 p* = 0.49). Cohort analysis within Project MinE and meta‐analysis of the cohorts did not reveal any differences (see Fig 4). In addition, other CN categories, including duplications of *SMN1* or *SMN2* (lbl; *SMN1 p* = 0.55 and *SMN2 p* = 0.71; binomial multivariate logistic regression; *SMN1 p* = 0.73 and *SMN2 p* = 0.83) or deletions (lbl; *SMN1 p* = 0.69 and *SMN2 p* = 0.22; binomial multivariate logistic regression; SMN1 *p* = 0.98 and *SMN2 p* = 0.39) did not associate with ALS.

### ****SMN**** ***CNs as a Modifier for ALS***

**FIGURE 5:**
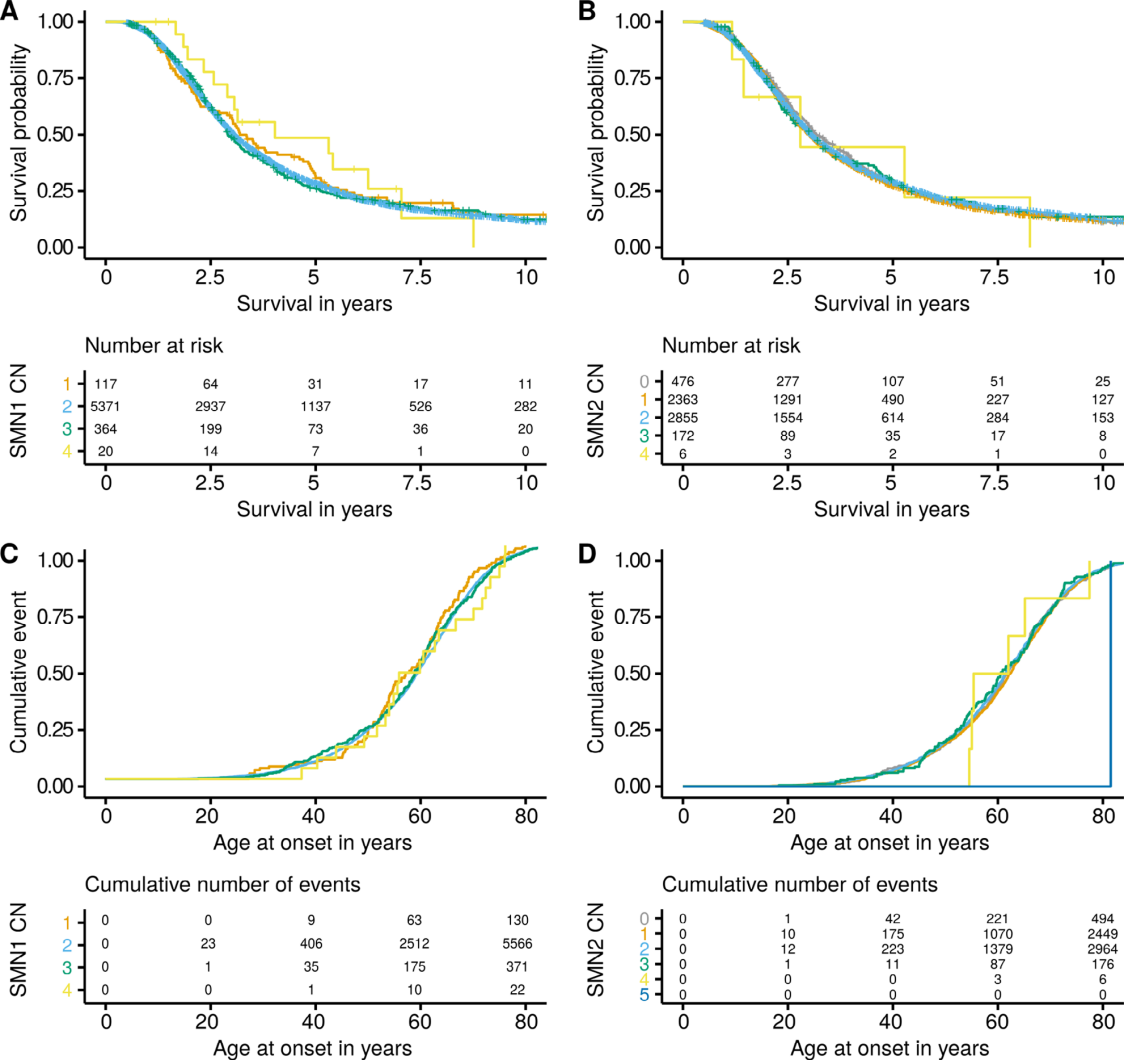
Survival and onset of patients, stratified by SMN CN. Kaplan Meier survival curves and risk table for (A) *SMN1* and (B) *SMN2* CNs. Kaplan Meier age at onset curves and cumulative events table for (C) *SMN1* and (D) *SMN2*. CN = copy number; SMN = survival of motor neuron.

As the CN of *SMN* has been proposed as a disease modifier, we also investigated the effect on age at onset and survival.^10^, ^15^ For this, we performed both a Cox regression and a RP analysis,^28^ while correcting for covariates. This revealed no significant association with survival for *SMN1* (Cox hazard ratio [HR] = 1.00, 95% confidence interval [CI] = 0.91–1.11, *p* = 0.89; RP HR = 0.98, 95% CI = 0.84–1.14, *p* = 0.78) or *SMN2* (Cox HR = 1.01, 95% CI = 0.97–1.05, *p* = 0.67; RP HR = 1.04, 95% CI = 0.97–1.12, *p* = 0.23; Fig 5A, B). Meta analyzing the results in the individual cohorts with a random effect model revealed no significant associations for *SMN1* (Cox HR = 1.00, 95% CI = 0.90–1.10, *p* = 0.96; RP HR = 0.95, 95% CI = 0.80–1.13, *p* = 0.59) or *SMN2* (cox HR = 1.01, 95% CI = 0.95–1.07, *p* = 0.73; RP HR = 1.04, 95% CI = 0.95–1.13, *p* = 0.43). Power analysis showed that our cohort had an 80% power to detect a 25% change in expected hazard for *SMN1* deletion (~ 9 months), 16% for *SMN1* duplication (~ 6 months), 9% for *SMN2* deletion (~ 3 months), and 22% for *SMN2* duplications (~ 9 months).

Similarly, we investigated the effect on onset, while correcting for covariates. Neither *SMN1* nor *SMN2* showed a significant association with onset at the level of the whole cohort (*SMN1*; Cox HR = 0.98, 95% CI = 0.90–1.06, *p* = 0.63, RP HR = 0.98, 95% CI = 0.85–1.13, *p* = 0.75; *SMN2*; Cox HR = 1.00, 95% CI = 0.97–1.04, *p* = 0.86, RP HR = 1.01, 95% CI = 0.95–1.08, *p* = 0.63) or by the cohort analysis (see Fig 5C, D). Likewise, meta analyzing using a random effect model revealed no significant associations for *SMN1* (Cox HR = 0.97, 95% CI = 0.89–1.06, *p* = 0.49; RP HR = 0.94, 95% CI = 0.75–1.17, *p* = 0.57) or *SMN2* (Cox HR = 1.00, 95% CI = 0.94–1.07, *p* = 0.85; RP HR = 1.01, 95% CI = 0.91–1.13, *p* = 0.83). Power analysis for the age at onset analysis showed we had an 80% power to detect a 21% change in expected hazard for *SMN1* deletion, 14% for *SMN1* duplication, 7% for *SMN2* deletion, and 19% for *SMN2* duplications.

## Discussion

Our study, in a large cohort of patients with ALS for which *SMN1* and *SMN2* CNs have been assessed, shows that no association can be found between the CN of the *SMN* genes and ALS risk or disease severity. This is in line with some previously published studies, but inevitably also contradicts some previous findings in much smaller studies.^6^, ^7^, ^8^, ^9^, ^10^, ^11^, ^12^, ^13^, ^14^, ^15^ Given that our study is currently by far the largest available, includes samples spread out over a wide range of geographical areas, is well powered, and subgroup analyses did not reveal any associations, this study provides conclusive evidence that *SMN* genes do not have a role in ALS pathogenesis through SMN gene CN. Previous studies reported an increased risk of ALS up to 2‐fold to 5‐fold for carriers with only one copy of *SMN1*, which represent carrier status of SMA.^9^, ^12^, ^13^ Our study clearly shows that SMA carriers, representing ~ 2% of the population, are not at an increased risk of developing ALS. The CN distribution in our control samples were consistent with an independent large control cohort, further validating our approach.^27^

WGS data identify a multitude of genomic variations, including single point variants, small insertions, deletions, and structural variations. Additionally, an increasing amount of reliable bioinformatic tools become available for WGS data to assess complex genomic regions like, for example, repeat expansions or genomic duplications like the *SMN* region, allowing to extract new information from WGS data with unprecedented ease and speed, without the necessity of additional wet lab experiments. A recent study evaluating the reliability of wet lab testing of the C9orf72 repeat across multiple laboratories, showed a low concordance between different laboratories, highlighting the difficulties of wet lab testing and accompanying result interpretations.^29^ It has recently been shown that C9orf72 calls made from WGS data were more reliable than their wet lab counterparts.^23^ The power of WGS is that it offers a one‐stop solution for all the known genetically relevant ALS information, including genetic variation in complex regions.

Recently, SMA became a treatable disease currently with 3 treatment options. The first option is the use of chronic intrathecal antisense oligonucleotide therapy, that aims to convert *SMN2* gene products that lack exon 7 into full length SMN1 protein.^19^ The second option is a single dose viral gene replacement therapy that adds an extra copy of *SMN1* into the patient's genome.^20^ In addition, compound therapies are being developed to increase the expression of the SMN locus. These breakthroughs in SMA could be important for ALS as well. The SMN1 protein plays important roles in RNA metabolism, which has been implicated in ALS as well. However, the motor neuron death cascades seem to be distinct in both diseases.^30^, ^31^ Given our findings and the differences at genetic and molecular level that led to motor neuron death in ALS and SMA, current SMA treatment options may not be beneficial for patients with ALS.

Although our genetic findings show that SMN CN is not involved in ALS, it does not entirely rule out that changes in protein levels contribute to motor neuron degeneration in ALS. SMN protein levels are not solely determined by the CN state of the *SMN* genes, many processes influence protein level, including mRNA transcription, translation efficiency, post translational modification, and protein aggregation and sequestration. CN levels of *SMN1* and *SMN2* correlate with mRNA levels in blood, spinal cord, and cerebellum, as previously shown.^32^, ^33^ Protein levels do so to a lesser extent in blood and cerebellum, and not in the spinal cord.^32^, ^34^ Taken together these results show protein levels cannot be reliably predicted on *SMN* CN state alone. Furthermore, our findings do not rule out that overexpression of *SMN* to levels above the physiological range can have beneficial effects in protecting motor neurons in ALS. Indeed, previous work showed that motor neurons with the same genetic background display a wide heterogeneity of *SMN* expression and that mostly the motor neurons with a low level of *SMN* are vulnerable for motor neuron death.^35^ Additionally, they showed that using compounds that increase SMN protein levels, through *SMN1* expression promotion, but not *SMN2*, were beneficial for the survival of motor neurons, not only in SMA but also in an ALS context and in healthy controls.^35^

This study has some limitations. Project MinE is not a population‐based study, but a multicenter study in which data and samples from different centers and countries are included. Samples are mostly from people of European descent. This could potentially limit the external validity to a wider, more diverse population and lead to population stratification. However, Project MinE explicitly aims to include balanced case control cohorts resulting in control samples not being significantly different from previously published data sets.^21^ This, together with the large sample size and multivariate analyses, therefore add to the credibility of these results. Last, given the complexity of the SMN locus, we did not investigate the role of point mutations and small indel in the *SMN* genes in the context of ALS, as these mutations could also lead to a loss‐of‐function, like *SMN1* gene deletions. In the light of our current results and the fact that they represent only 5% of *SMN1* mutations, chances are small that these do play a role in ALS.^17^

In summary, in our well‐powered study, using highly reliable and validated SMN calls, there was no association of *SMN1* or *SMN2* CNs with the risk of ALS or ALS disease severity. This suggests that changing SMN expression levels in the physiological range may not modify the progression of ALS. This is an important finding in the light of emerging therapies targeted at SMN deficiencies.

## Author Contributions

M.M., P.V.D., and J.V. contributed to the conception and design of the study. All authors contributed to the acquisition and analysis of data. M.M., P.V.D., J.V., X.C., and M.A.E. contributed to drafting the text and preparing the figures.

## Potential Conflicts of Interest

X.C. and M.A.E. are employees of Illumina Inc.

## Data Availability

Data can be accessed through EGAC00001000703. The authors declare that all the other data supporting the findings of this study are available within the article, its Supplementary Information files, or from the corresponding author upon reasonable request.

## Supporting information


**Table S1** Phenotype by country

**Table S2** Frequency of *SMN* genes in different populations

**Table S3** Frequency of the *SMN1* gene in different countries within Project MinE

**Table S4** Frequency of *SMN2* in different countries within Project MinE
