# Supplementary material for: The Effect of SMN Gene Dosage on ALS Risk and Disease Severity
Source: Ann Neurol. 2021 Jan 15;89(4):686–97. doi: 10.1002/ana.26009 (PMC8048961; doi:10.1002/ana.26009)
Supplement: Supplementary file 1 — Table S1 Phenotype by country [file ANA-89-686-s001.docx]

## **Supplementary table S1 Phenotype by country**

BE=Belgium, CH=Swiss, ES=Spain, FR=France, GB=United Kingdom, IE=Ireland, IL=Israel, IT=Italy, NL=The Netherlands, PT=Portugal, SE=Sweden, TR=Turkey, US=United States

|  | ALS (%) | | Control (%) | |  |
| --- | --- | --- | --- | --- | --- |
| Project Mine | 6375 | (72.55) | 2412 | (27.45) |  |
| BE | 547 | (75.14) | 181 | (24.86) |  |
| CH | 53 | (100) | 0 | (0) |  |
| ES | 378 | (70.52) | 158 | (29.48) |  |
| FR | 210 | (84.68) | 38 | (15.32) |  |
| GB | 1498 | (77.34) | 439 | (22.66) |  |
| IE | 465 | (66.52) | 234 | (33.48) |  |
| IL | 104 | (100) | 0 | (0) |  |
| IT | 61 | (100) | 0 | (0) |  |
| NL | 1789 | (63.26) | 1039 | (36.74) |  |
| PT | 59 | (81.94) | 13 | (18.06) |  |
| SE | 201 | (64.42) | 111 | (35.58) |  |
| TR | 603 | (82.04) | 132 | (17.96) |  |
| US | 407 | (85.86) | 67 | (14.14) |  |
| Sex male | 3815 | (59.84) | 1272 | (52.74) | <0.01 |
| BE | 331 | (60.51) | 107 | (59.12) | 0.71 |
| CH | 35 | (66.04) |  |  |  |
| ES | 223 | (58.99) | 78 | (49.37) | 0.05 |
| FR | 122 | (58.1) | 18 | (47.37) | 0.29 |
| GB | 912 | (60.88) | 173 | (39.41) | <0.01 |
| IE | 281 | (60.43) | 138 | (58.97) | 0.77 |
| IL | 59 | (56.73) |  |  |  |
| IT | 34 | (55.74) |  |  |  |
| NL | 1062 | (59.36) | 612 | (58.9) | 0.84 |
| PT | 31 | (52.54) | 6 | (46.15) | 0.91 |
| SE | 117 | (58.21) | 51 | (45.95) | 0.05 |
| TR | 356 | (59.04) | 63 | (47.73) | 0.02 |
| US | 252 | (61.92) | 26 | (38.81) | <0.01 |
| C9orf72 expansion | 377 | (5.91) | 7 | (0.29) | <0.01 |
| BE | 33 | (6.03) | 0 | (0) | <0.01 |
| CH | 5 | (9.43) |  |  |  |
| ES | 13 | (3.44) | 0 | (0) | 0.04 |
| FR | 11 | (5.24) | 1 | (2.63) | 0.78 |
| GB | 98 | (6.54) | 4 | (0.91) | <0.01 |
| IE | 43 | (9.25) | 0 | (0) | <0.01 |
| IL | 4 | (3.85) |  |  |  |
| IT | 1 | (1.64) |  |  |  |
| NL | 118 | (6.6) | 2 | (0.19) | <0.01 |
| PT | 2 | (3.39) | 0 | (0) | 1.00 |
| SE | 21 | (10.45) | 0 | (0) | <0.01 |
| TR | 0 | (0) | 0 | (0) | <0.01 |
| US | 28 | (6.88) | 0 | (0) | 0.05 |
| Onset spinal | 4226 | (66.29) |  |  |  |
| BE | 362 | (66.18) |  |  |  |
| CH | 37 | (69.81) |  |  |  |
| ES | 281 | (74.34) |  |  |  |
| FR | 149 | (70.95) |  |  |  |
| GB | 939 | (62.68) |  |  |  |
| IE | 314 | (67.53) |  |  |  |
| IL | 75 | (72.12) |  |  |  |
| IT | 48 | (78.69) |  |  |  |
| NL | 1145 | (64) |  |  |  |
| PT | 43 | (72.88) |  |  |  |
| SE | 135 | (67.16) |  |  |  |
| TR | 421 | (69.82) |  |  |  |
| US | 277 | (68.06) |  |  |  |
| Clinical data available |  |  |  |  |  |
| survival | 5926 | (92.96) |  |  |  |
