# Supplementary material for: The Effect of SMN Gene Dosage on ALS Risk and Disease Severity
Source: Ann Neurol. 2021 Jan 15;89(4):686–97. doi: 10.1002/ana.26009 (PMC8048961; doi:10.1002/ana.26009)
Supplement: Supplementary file 2 — Table S2 Frequency of SMN genes in different populations [file ANA-89-686-s004.docx]

## **Supplementary table S2 Frequency of *SMN* genes in different populations**

|  |  | SMN1 | | | | | | | | | | SMN2 | | | | | | | | | |
| --- | --- | --- | --- | --- | --- | --- | --- | --- | --- | --- | --- | --- | --- | --- | --- | --- | --- | --- | --- | --- | --- |
|  | CN (%) | 0 | | 1 | | 2 | | 3 | | 4 | | 0 | | 1 | | 2 | | 3 | | 4 | |
| Project MinE | Ctrl | 0 | (0.0) | 47 | (0.0) | 2204 | (0.9) | 144 | (0.1) | 3 | (0.0) | 201 | (0.1) | 995 | (0.4) | 1136 | (0.5) | 61 | (0.0) | 5 | (0.0) |
|  | ALS | 0 | (0.0) | 132 | (0.0) | 5776 | (0.9) | 387 | (0.1) | 22 | (0.0) | 514 | (0.1) | 2537 | (0.4) | 3077 | (0.5) | 182 | (0.0) | 6 | (0.0) |
| Caucasian | Ctrl | 0 | (0.0) | 31 | (1.4) | 1994 | (91.7) | 150 | (6.9) | 0 | (0.0) |  |  |  |  |  |  |  |  |  |  |
| Netherlands | Ctrl | 0 | (0.0) | 23 | (2.3) | 926 | (94.1) | 35 | (3.6) | 0 | (0.0) | 78 | (7.9) | 372 | (37.8) | 486 | (49.4) | 46 | (4.7) | 2 | (0.2) |
|  | ALS | 0 | (0.0) | 16 | (1.9) | 771 | (91.0) | 60 | (7.1) | 0 | (0.0) | 62 | (7.3) | 329 | (38.8) | 416 | (49.1) | 39 | (4.6) | 1 | (0.1) |
| France | Ctrl | 0 | (0.0) | 13 | (2.1) | 593 | (95.5) | 15 | (2.4) | 0 | (0.0) | 52 | (8.4) | 239 | (38.5) | 321 | (51.7) | 9 | (1.4) | 0 | (0.0) |
|  | ALS | 0 | (0.0) | 36 | (6.0) | 529 | (88.2) | 35 | (5.8) | 0 | (0.0) | 54 | (9.0) | 234 | (39.0) | 305 | (50.8) | 7 | (1.2) | 0 | (0.0) |
| Hispanic | Ctrl | 0 | (0.0) | 12 | (0.9) | 1220 | (89.8) | 127 | (9.3) | 0 | (0.0) |  |  |  |  |  |  |  |  |  |  |
| Ashkenazi Jewish | Ctrl | 0 | (0.0) | 10 | (2.4) | 372 | (90.5) | 29 | (7.1) | 0 | (0.0) |  |  |  |  |  |  |  |  |  |  |
| Asian | Ctrl | 0 | (0.0) | 10 | (2.4) | 372 | (90.5) | 29 | (7.1) | 0 | (0.0) |  |  |  |  |  |  |  |  |  |  |
| Korea | Ctrl | 0 | (0.0) | 2 | (2.0) | 96 | (97.0) | 1 | (1.0) | 0 | (0.0) | 2 | (2.0) | 29 | (29.3) | 64 | (64.6) | 4 | (4.0) | 0 | (0.0) |
| China | Ctrl | 0 | (0.0) | 1 | (0.5) | 199 | (97.1) | 5 | (2.4) | 0 | (0.0) | 0 | (0.0) | 66 | (33.7) | 128 | (65.3) | 2 | (1.0) | 0 | (0.0) |
| African American | Ctrl | 0 | (0.0) | 14 | (1.0) | 688 | (51.1) | 644 | (47.8) | 0 | (0.0) |  |  |  |  |  |  |  |  |  |  |

## 
