# Supplementary material for: The Effect of SMN Gene Dosage on ALS Risk and Disease Severity
Source: Ann Neurol. 2021 Jan 15;89(4):686–97. doi: 10.1002/ana.26009 (PMC8048961; doi:10.1002/ana.26009)
Supplement: Supplementary file 3 — Table S3 Frequency of the SMN1 gene in different countries within Project MinE [file ANA-89-686-s002.docx]

## **Supplementary table S3 Frequency of the SMN1 gene in different countries within Project MinE**

|  |  | SMN1 | | | | | | | | | |
| --- | --- | --- | --- | --- | --- | --- | --- | --- | --- | --- | --- |
|  | CN (%) | 0 | | 1 | | 2 | | 3 | | 4 | |
| BE | Ctrl | 0 | (0.0) | 5 | (2.8) | 169 | (93.4) | 7 | (3.9) | 0 | (0.0) |
|  | ALS | 0 | (0.0) | 10 | (1.8) | 495 | (91.2) | 36 | (6.6) | 2 | (0.4) |
| IE | Ctrl | 0 | (0.0) | 6 | (2.6) | 215 | (93.1) | 10 | (4.3) | 0 | (0.0) |
|  | ALS | 0 | (0.0) | 5 | (1.1) | 434 | (93.9) | 21 | (4.5) | 2 | (0.4) |
| NL | Ctrl | 0 | (0.0) | 11 | (1.1) | 966 | (93.2) | 57 | (5.5) | 3 | (0.3) |
|  | ALS | 0 | (0.0) | 46 | (2.6) | 1612 | (90.7) | 112 | (6.3) | 7 | (0.4) |
| SE | Ctrl | 0 | (0.0) | 1 | (0.9) | 101 | (91.8) | 8 | (7.3) | 0 | (0.0) |
|  | ALS | 0 | (0.0) | 4 | (2.0) | 187 | (93.0) | 10 | (5.0) | 0 | (0.0) |
| US | Ctrl | 0 | (0.0) | 0 | (0.0) | 60 | (90.9) | 6 | (9.1) | 0 | (0.0) |
|  | ALS | 0 | (0.0) | 11 | (2.7) | 367 | (91.5) | 21 | (5.2) | 2 | (0.5) |
| GB | Ctrl | 0 | (0.0) | 13 | (3.0) | 394 | (90.8) | 27 | (6.2) | 0 | (0.0) |
|  | ALS | 0 | (0.0) | 26 | (1.7) | 1370 | (91.9) | 89 | (6.0) | 5 | (0.3) |
| ES | Ctrl | 0 | (0.0) | 3 | (1.9) | 142 | (89.9) | 13 | (8.2) | 0 | (0.0) |
|  | ALS | 0 | (0.0) | 9 | (2.5) | 330 | (90.9) | 24 | (6.6) | 0 | (0.0) |
| TR | Ctrl | 0 | (0.0) | 5 | (3.8) | 114 | (87.7) | 11 | (8.5) | 0 | (0.0) |
|  | ALS | 0 | (0.0) | 15 | (2.5) | 545 | (91.3) | 35 | (5.9) | 2 | (0.3) |
| PT | Ctrl | 0 | (0.0) | 1 | (7.7) | 11 | (84.6) | 1 | (7.7) | 0 | (0.0) |
|  | ALS | 0 | (0.0) | 1 | (1.8) | 53 | (93.0) | 3 | (5.3) | 0 | (0.0) |
| FR | Ctrl | 0 | (0.0) | 2 | (5.3) | 32 | (84.2) | 4 | (10.5) | 0 | (0.0) |
|  | ALS | 0 | (0.0) | 3 | (1.4) | 190 | (90.5) | 16 | (7.6) | 1 | (0.5) |
| IT | ALS | 0 | (0.0) | 0 | (0.0) | 58 | (95.1) | 3 | (4.9) | 0 | (0.0) |
| IL | ALS | 0 | (0.0) | 0 | (0.0) | 91 | (88.3) | 11 | (10.7) | 1 | (1.0) |
| CH | ALS | 0 | (0.0) | 2 | (3.8) | 44 | (84.6) | 6 | (11.5) | 0 | (0.0) |
