# Supplementary material for: The Effect of SMN Gene Dosage on ALS Risk and Disease Severity
Source: Ann Neurol. 2021 Jan 15;89(4):686–97. doi: 10.1002/ana.26009 (PMC8048961; doi:10.1002/ana.26009)
Supplement: Supplementary file 4 — Table S4 Frequency of SMN2 in different countries within Project MinE [file ANA-89-686-s003.docx]

## **Supplementary table S4 Frequency of SMN2 in different countries within Project MinE**

|  |  | SMN2 | | | | | | | | | | | |
| --- | --- | --- | --- | --- | --- | --- | --- | --- | --- | --- | --- | --- | --- |
|  | CN (%) | 0 | | 1 | | 2 | | 3 | | 4 | | 5 | |
| BE | Ctrl | 15 | (8.3) | 67 | (37.0) | 93 | (51.4) | 6 | (3.3) | 0 | (0.0) | 0 | (0.0) |
|  | ALS | 50 | (9.2) | 217 | (40.0) | 266 | (49.0) | 10 | (1.8) | 0 | (0.0) | 0 | (0.0) |
| IE | Ctrl | 15 | (6.5) | 113 | (48.9) | 100 | (43.3) | 2 | (0.9) | 1 | (0.4) | 0 | (0.0) |
|  | ALS | 41 | (8.9) | 185 | (40.0) | 229 | (49.6) | 7 | (1.5) | 0 | (0.0) | 0 | (0.0) |
| NL | Ctrl | 88 | (8.5) | 445 | (42.9) | 480 | (46.3) | 21 | (2.0) | 3 | (0.3) | 0 | (0.0) |
|  | ALS | 163 | (9.2) | 711 | (40.0) | 837 | (47.1) | 66 | (3.7) | 0 | (0.0) | 0 | (0.0) |
| SE | Ctrl | 2 | (1.8) | 51 | (46.4) | 55 | (50.0) | 2 | (1.8) | 0 | (0.0) | 0 | (0.0) |
|  | ALS | 14 | (7.0) | 73 | (36.3) | 109 | (54.2) | 5 | (2.5) | 0 | (0.0) | 0 | (0.0) |
| US | Ctrl | 7 | (10.6) | 26 | (39.4) | 30 | (45.5) | 3 | (4.5) | 0 | (0.0) | 0 | (0.0) |
|  | ALS | 35 | (8.7) | 181 | (45.1) | 177 | (44.1) | 8 | (2.0) | 0 | (0.0) | 0 | (0.0) |
| GB | Ctrl | 42 | (9.7) | 177 | (40.8) | 195 | (44.9) | 20 | (4.6) | 0 | (0.0) | 0 | (0.0) |
|  | ALS | 113 | (7.6) | 622 | (41.7) | 710 | (47.7) | 41 | (2.8) | 3 | (0.2) | 0 | (0.0) |
| ES | Ctrl | 18 | (11.4) | 51 | (32.3) | 86 | (54.4) | 3 | (1.9) | 0 | (0.0) | 0 | (0.0) |
|  | ALS | 24 | (6.6) | 156 | (43.0) | 173 | (47.7) | 8 | (2.2) | 2 | (0.6) | 0 | (0.0) |
| TR | Ctrl | 7 | (5.4) | 47 | (36.2) | 73 | (56.2) | 2 | (1.5) | 1 | (0.8) | 0 | (0.0) |
|  | ALS | 31 | (5.2) | 204 | (34.2) | 340 | (57.0) | 21 | (3.5) | 1 | (0.2) | 0 | (0.0) |
| PT | Ctrl | 2 | (15.4) | 3 | (23.1) | 8 | (61.5) | 0 | (0.0) | 0 | (0.0) | 0 | (0.0) |
|  | ALS | 3 | (5.3) | 20 | (35.1) | 33 | (57.9) | 1 | (1.8) | 0 | (0.0) | 0 | (0.0) |
| FR | Ctrl | 5 | (13.2) | 15 | (39.5) | 16 | (42.1) | 2 | (5.3) | 0 | (0.0) | 0 | (0.0) |
|  | ALS | 25 | (11.9) | 87 | (41.4) | 89 | (42.4) | 9 | (4.3) | 0 | (0.0) | 0 | (0.0) |
| IT | ALS | 5 | (8.2) | 20 | (32.8) | 35 | (57.4) | 1 | (1.6) | 0 | (0.0) | 0 | (0.0) |
| IL | ALS | 9 | (8.7) | 42 | (40.8) | 48 | (46.6) | 4 | (3.9) | 0 | (0.0) | 0 | (0.0) |
| CH | ALS | 1 | (1.9) | 19 | (36.5) | 31 | (59.6) | 1 | (1.9) | 0 | (0.0) | 0 | (0.0) |

## 
